# Investigation of serum agouti-related peptide levels in women with polycystic ovary syndrome

**DOI:** 10.1590/1806-9282.20241836

**Published:** 2025-06-16

**Authors:** Gülsüm Kırılmaz, İbrahim Kale

**Affiliations:** 1 Umraniye Training and Research Hospital, Department of Obstetrics and Gynecology – Ümraniye, Turkey.

**Keywords:** Agouti-related peptide, Hyperandrogenism, Insulin resistance, Polycystic ovary syndrome

## Abstract

**OBJECTIVE:**

Agouti-related peptide, secreted by the terminals of agouti-related peptide neurons in the hypothalamus and released into the systemic circulation, has been implicated in regulating food intake, obesity, and the development of insulin resistance. Therefore, the aim of this study was to evaluate serum agouti-related peptide levels in women with polycystic ovary syndrome.

**METHODS:**

This cross-sectional, case–control study included 88 women with polycystic ovary syndrome and 88 age- and body mass index-matched controls. Serum agouti-related peptide levels were quantified using commercial enzyme-linked immunosorbent assay kits. First, serum agouti-related peptide levels were compared between the control and polycystic ovary syndrome groups, followed by comparisons between the subgroups.

**RESULTS:**

The median serum agouti-related peptide level was 331.1 ng/L in the polycystic ovary syndrome group and 362 ng/L in the control group (p=0.994). Both the polycystic ovary syndrome and control groups were further stratified by body mass index into normal weight and overweight subgroups. Serum agouti-related peptide levels were similar across all the four subgroups (p=0.717). Additionally, the polycystic ovary syndrome group was divided into five subgroups based on polycystic ovary syndrome phenotype and compared with the control group regarding serum agouti-related peptide levels. Serum agouti-related peptide levels were comparable across the five subgroups (p=0.267).

**CONCLUSIONS:**

Serum agouti-related peptide levels were comparable between the polycystic ovary syndrome and non-polycystic ovary syndrome groups and in the subgroup analyses. Although the sample size was insufficient to draw definitive conclusions, the results suggest that serum agouti-related peptide levels are unlikely to play a significant role in the pathophysiology of polycystic ovary syndrome or be directly influenced by the condition.

## INTRODUCTION

Polycystic ovary syndrome (PCOS) is a heterogeneous endocrine disorder that affects approximately 8–13% of women of reproductive age and 6.3–9.8% of adolescents^1,2^. The revised Rotterdam criteria are used to diagnose PCOS. According to these criteria, PCOS is diagnosed when at least two of the following three conditions are present: oligo-anovulation, ­clinical or biochemical hyperandrogenism, and polycystic ovary ­morphology^3^. Based on the association of these disorders, the National Institutes of Health PCOS workshop in 2012 reported four distinct subgroups of PCOS (phenotypes A, B, C, and D). Phenotype A is often referred to as the complete PCOS phenotype. Both phenotypes A and B are commonly referred to as classic PCOS^4^. It has been reported that PCOS phenotypes may vary due to disruptions in glucose and lipid metabolism associated with the condition^5^. Women with ­classic PCOS tend to be more hirsute and obese, exhibit a more irregular menstrual pattern, and are more likely to have insulin resistance, with an increased risk of metabolic syndrome compared to those with ovulatory or non-hyperandrogenic phenotypes (C and D)^3^.

The precise etiology of PCOS remains unclear. However, it is thought to involve a combination of genetic predisposition, hypothalamic and ovarian dysfunction, insulin resistance, excessive androgen secretion, and obesity-related factors contributing to its pathophysiology^6^. A recent meta-analysis revealed that amenorrhea was the primary menstrual pattern characteristic of distinguishing girls with PCOS from those with hypothalamic–pituitary–ovarian axis immaturity^7^. Adult women with PCOS not only experience menstrual irregularities and infertility but also have an increased risk of developing diabetes, obesity, metabolic syndrome, cardiovascular diseases, and even uterine cancer^8^. Interestingly, it has been suggested that histological abnormalities persist in the endometrium despite progesterone supplementation in PCOS and that this is associated with the circulating androgen and insulin levels^9^.

Agouti-related peptide (AGRP), first identified by Ollmann et al. in 1997, is one of the most potent appetite stimulators in the hypothalamus^10^. In the central nervous system (CNS), AGRP is secreted from the terminals of AGRP neurons in the hypothalamus, which enters the systemic circulation by ­crossing the blood–brain barrier^11-13^. Peripherally, AGRP is expressed in the adrenal cortex^14^.

In the CNS, AGRP neurons promote feeding by ­inhibiting proopiomelanocortin (POMC) neurons and sending projections to various brain regions^15,16^. Additionally, studies have shown that insulin signaling in AGRP neurons helps regulate meal size, suggesting that AGRP may play a role in preventing the development of obesity and insulin resistance^17,18^. Given that insulin resistance is the primary disorder in PCOS, we aimed to investigate how serum AGRP levels are affected in PCOS by comparing serum AGRP levels in women with and ­without the condition.

## METHODS

This cross-sectional, case–control study was conducted at the Department of Obstetrics and Gynecology, Umraniye Training and Research Hospital, Istanbul, Turkey, between December 2023 and May 2024. The PCOS group consisted of 88 women diagnosed with PCOS aged between 18 and 39 years. PCOS was diagnosed based on the revised Rotterdam criteria. A diagnosis of PCOS was established when at least two of the following three criteria were present: oligo-anovulation, clinical or biochemical hyperandrogenism, and polycystic ovary ­morphology^8^. A free androgen index was used to diagnose hirsutism^19^. The free androgen index is calculated as 100× (total testosterone/sex hormone-binding protein)^8^. After PCOS diagnosis, participants were classified into one of the four different phenotypes: those with oligo/amenorrhea, hyperandrogenism, and ultrasound findings of polycystic ovaries were included in phenotype A; those with oligo/amenorrhea and hyperandrogenism were included in phenotype B; those with ultrasound findings of polycystic ovaries and hyperandrogenism were included in phenotype C; and those with ultrasound findings of ­polycystic ovaries and oligo/amenorrhea were included in phenotype D^3^. The control group comprised 88 healthy women matched to the PCOS group for age and body mass index (BMI). Smokers, alcohol consumers, women with chronic diseases or using any medication, those under 18 or over 39 years of age, and women with a BMI below 18.5 kg/m^2^ or above 40 kg/m^2^ were not included in the study.

For serum AGRP levels and other blood tests, blood samples were collected from participants after 8 h of fasting on the morning of the 2nd or 3rd day of their menstrual cycle via antecubital venipuncture. Blood samples collected for AGRP were kept at room temperature for 20 min, then centrifuged at 2,500 RPM for 20 min. After centrifugation, the remaining serum portion in the biochemical tube was transferred to Eppendorf tubes and stored at −80°C until the analysis day. AGRP levels in serum samples were studied with the Human AGRP ELISA Kit (SunRed Biotechnology Company, catalog number: 201-12-1479). The Human AGRP ELISA Kit measurement range used in the study was between 5 ng/L and 1,500 ng/L, and the kit's sensitivity was 4.776 ng/L.

First, the PCOS and control groups, then the subgroups created according to BMI, and the groups created according to PCOS phenotypes were compared regarding serum AGRP levels.

The Istanbul Umraniye Training and Research Hospital's Local Ethics Committee approved this study (approval number: B.10.1.TKH.4.34.H.GP.0.01/390, date: 03/11/2023). The study was conducted by following the principles of the Declaration of Helsinki and the country's ethical standards. Informed and written consent was obtained from all participants.

### Statistical analysis

Since there is no literature study investigating serum AGRP levels in women with PCOS, the power analysis of this study was performed according to the sample size estimation method using simple random sampling with a known universe. The sample size estimation formula in the simple random sampling with a known universe is $\text{n}={\text{Nt}}^{2}\text{pq}/{\text{d}}^{2}(\text{N}-1)+{\text{t}}^{2}\text{pq}$ (N: number of individuals in the universe, n: number of individuals to be sampled, p: frequency of occurrence of the event under study, q: frequency of non-occurrence of the event under study, t: theoretical value found from the t table at a certain degree of freedom and the determined level of error, and d: deviation desired to be made according to the frequency of occurrence of the event). A total of 939 women diagnosed with PCOS applied to our outpatient clinic between December 1, 2023, and May 31, 2024. According to the abovementioned formula, the number of participants in each group was determined as 55 to obtain 80% power at the α=0.05 level. Considering the possible dropouts during the study, 88 participants were included in each group. Since there was no dropout in the study, the study was conducted with 176 participants (88 in the PCOS group and 88 in the control group).

Statistical analyses were performed using the Statistical Package for the Social Sciences version 29 program (SPSS Inc.; Chicago, IL, USA). During the data analysis, quantitative variables were presented as mean, standard deviation, median, minimum, and maximum values, and qualitative variables were presented with descriptive statistical methods, such as frequency and percentage. The conformity of the data to normal distribution was assessed with the Shapiro-Wilk test and box plot graphics. An independent t-test was used to compare variables showing normal distribution between the two groups, and a Mann-Whitney U test was used to compare variables that did not show normal distribution between the two groups. Variables showing non-normal distribution between the two groups were analyzed using the Kruskal-Wallis test. While the correlation coefficient was used to determine the relationship between quantitative variables, the chi-square test was used for group comparisons of qualitative data. Spearman's correlation analysis determined the relationship between PCOS-related parameters and serum AGRP. Statistical significance was accepted as p<0.05 for all variables.

## RESULTS

Age, BMI, waist and hip circumferences, gravida, and parity were similar between the PCOS and control groups (p>0.05 for all). Both groups were also comparable in terms of systolic, diastolic, and mean arterial pressures (p>0.05 for all) (Table 1).

**Table 1:** Comparison of polycystic ovary syndrome and control groups in terms of demographic characteristics.

			PCOS group (n=88), median (min–max), n (%)	Control group (n=88), median (min–max), n (%)	p
Age (years)			26 (18–37)	28 (18–39)	0.061^a^
Body mass index (kg/m^2^)			25.1 (18–39.6)	24.7 (18.1–39.3)	0.405^a^
Waist circumference (mm)			92.5 (63–140)	84 (62–156)	0.710^a^
Hip circumference (mm)			106 (89–162)	106 (92–162)	0.810^a^
Gravida			0 (0–5)	0 (0–7)	0.086^a^
Parity		Nulliparous	59 (67)	48 (54.5)	0.061^b^
		Multiparous	29 (33)	40 (45.5)	
Systolic blood pressure (mmHg)			100 (90–120)	102.5 (90–123)	0.105^a^
	Diastolic blood pressure (mmHg)		61 (50–80)	60 (55–82)	0.773^a^
	Mean arterial pressure (mmHg)		76 (63–90)	76 (68–95)	0.557^a^

a Mann-Whitney U test;

b chi-square test; PCOS: polycystic ovary syndrome.

Both groups were similar regarding fasting blood glucose, fasting insulin, Homeostasis Model Assessment of Insulin Resistance (HOMA-IR), and hemoglobin A1c (HbA1c) ­levels (p>0.05 for all). Free testosterone, total testosterone, and free androgen index were statistically significantly higher in the PCOS group compared to the control group, while sex hormone-binding globulin was statistically significantly lower (p=0.001, p<0.001, p<0.001, and p=0.002, respectively). Dehydroepiandrostenedione (DHEA) and dehydroepiandrostenedione sulfate (DHEAS) levels were statistically significantly higher in the PCOS group compared to the control group (p<0.001 and p<0.001, respectively). While estradiol, prolactin, and thyroid-stimulating hormone (TSH) levels were similar in both groups, the follicle-stimulating hormone (FSH) level was statistically significantly lower and the ­luteinizing hormone (LH) level was statistically significantly higher in the PCOS group compared to the control group (p=0.303, p=0.201, p=0.649, p=0.011, and p<0.001, respectively). The median serum AGRP level was 331.1 ng/L in the PCOS group and 362 ng/L in the control group (p=0.994) (Table 2).

**Table 2:** Comparison of polycystic ovary syndrome and control groups in terms of laboratory results.

	PCOS group (n=88), median (min–max), mean±SD, n (%)	Control group (n=88), median (min–max), mean±SD, n (%)	p
Fasting blood glucose (mg/dL)	85 (65–104)	85 (74–105)	0.859^a^
Fasting insulin (μU/mL)	8.7 (3.9–13)	8.3 (2.6–12.8)	0.702^a^
HOMA-IR	1.8 (0.8–2.4)	1.8 (0.6–2.5)	0.957^a^
HbA1c (%)	5.3 (4.3–5.6)	5.4 (4.8–5.7)	0.807^a^
Free testosterone (pg/mL)	2.9 (1.2–12)	2.5 (0.9–8)	0.001^a^
Total testosterone (ng/dL)	41.2 (9.5–107)	29.2 (8.2–48)	<0.001^a^
Sex hormone-binding globulin (ng/dL)	1,110.4 (230.7–5,768.4)	1,326.7 (461.4–5,364.6)	0.002^a^
Free androgen index	3.1 (0.4–32.1)	2 (0.2–6.2)	<0.001^a^
Dehydroepiandrosterone (μg/dL)	17 (3.7–48)	9 (2.8–16.7)	<0.001^a^
Dehydroepiandrosterone sulfate (μg/dL)	307.5 (87.4–977)	245 (37–339)	<0.001^a^
Follicle-stimulating hormone (IU/L)	5.7 (1.3–9)	6.1 (2.5–12.4)	0.011^a^
Luteinizing hormone (IU/L)	9.1 (2.4–25.7)	5.7 (1.9–19.4)	<0.001^a^
Estradiol (ng/L)	45.9 (12.7–303)	42.6 (3.1–403)	0.303^a^
Prolactin (μg/L)	17.6 (6–53.4)	16.4 (4.4–24.1)	0.201^a^
Thyroid-stimulating hormone (mIU/L)	2.1 (0.6–5.1)	1.8 (0.6–5.1)	0.649^a^
Agouti-related peptide (ng/L)	331.1 (21.1–1,650.6)	362 (3–1,496.8)	0.994^a^

a Mann-Whitney U test.

PCOS: polycystic ovary syndrome; SD: standard deviation; HOMA-IR: Homeostasis Model Assessment of Insulin Resistance; HbA1c: hemoglobin A1c.

Both groups were stratified by BMI into normal weight and overweight categories. The serum AGRP levels were 339.1 ng/L in the normal-weight control group, 449.7 ng/L in the overweight control group, 439.8 ng/L in the normal-weight PCOS group, and 314.6 ng/L in the overweight PCOS group (p=0.717). The PCOS group was further divided into four subgroups A (n=14), B (n=11), C (n=8), and D (n=55) ­according to the phenotype and compared with the control group in terms of serum AGRP levels. Serum AGRP levels were similar across the five groups (p=0.267).

Spearman's correlation analysis was performed to understand the correlation between PCOS-related parameters and serum AGRP levels. No significant correlation was found between any parameter associated with PCOS and serum AGRP (Table 3).

**Table 3:** Correlation analysis of polycystic ovary syndrome-related parameters with serum agouti-related peptide levels.

Serum AGRP levels		
	r	p
Age (years)	0.09	0.403
Body mass index (kg/m^2^)	-0.117	0.276
Waist circumference (mm)	-0.093	0.388
Hip circumference (mm)	-0.139	0.195
Gravida	-0.129	0.230
Systolic blood pressure (mmHg)	0.068	0.530
Diastolic blood pressure (mmHg)	-0.066	0.542
Mean arterial pressure (mmHg)	-0.023	0.829
Fasting blood glucose (mg/dL)	-0.123	0.253
Fasting insulin (μU/mL)	-0.049	0.649
HOMA-IR	-0.114	0.291
HbA1c (%)	-0.041	0.701
Free testosterone (pg/mL)	0.067	0.538
Total testosterone (ng/dL)	0.104	0.337
Sex hormone-binding globulin (ng/dL)	-0.073	0.499
Free androgen index	0.124	0.251
Dehydroepiandrosterone (μg/dL)	-0.083	0.443
Dehydroepiandrosterone sulfate (μg/dL)	-0.002	0.983
Follicle-stimulating hormone (IU/L)	0.043	0.689
Luteinizing hormone (IU/L)	-0.125	0.247
Estradiol (ng/L)	0.015	0.889
Prolactin (μg/L)	0.010	0.923
Thyroid-stimulating hormone (mIU/L)	-0.071	0.513

Spearman's correlation. AGRP: agouti-related peptide; HOMA-IR: Homeostasis Model Assessment of Insulin Resistance; HbA1c: hemoglobin A1c.

## DISCUSSION

In this study, we examined serum AGRP levels in ­individuals with PCOS. Initially, we hypothesized that serum AGRP ­levels would be elevated in the PCOS group compared to the non-PCOS group. However, contrary to our hypothesis, no significant difference in AGRP levels was observed between the two groups.

The hypothalamic POMC and AGRP neurons have been identified as the key targets of leptin and insulin signaling in the CNS. Numerous studies have sought to delineate the individual contributions of the various components within the primary pathways mediating leptin and insulin's central effects^20^. Tsaousidou et al. showed in their experimental study that constitutive c-Jun N-terminal kinase (JNK) activation in the AGRP neurons of the hypothalamus caused weight gain in mice due to hyperphagia. JNK activation increased the spontaneous action potential firing of AGRP neurons and caused neuronal and systemic leptin resistance. In contrast to JNK activation, IκB kinase 2 (IKK2) activation blunted insulin signaling in AGRP neurons and impaired systemic glucose homeostasis^18^. Similarly, Steculorum et al. showed that acutely activating AGRP neurons primarily impairs systemic insulin sensitivity by inhibiting insulin-stimulated glucose uptake in brown adipose tissue^21^. In a different study, Dodd et al. reported that insulin signaling in AGRP neurons regulates meal size, limiting glucose excursions and insulin resistance^17^. A recent study showed that the upregulation of spliced X-box binding protein 1 (Xbp1s) in AGRP neurons reverses diet-induced obesity and improves leptin and insulin resistance. The authors demonstrated that the constitutive expression of Xbp1s in AGRP neurons enhances insulin sensitivity and glucose tolerance^22^.

While AGRP neuronal activity in the CNS has been thoroughly investigated, the literature on circulating AGRP levels is quite limited. AGRP is secreted from the terminals of AGRP neurons in the hypothalamus and can cross the blood–brain barrier into the systemic circulation^12,13^. Peripheral AGRP is believed to be predominantly expressed in the adrenal cortex of both humans and animals, where it likely serves a paracrine function^14^. However, since bilateral adrenalectomy does not significantly affect serum AGRP levels in rats, the adrenal cortex is unlikely to be the primary source of serum AGRP^13^. Consequently, serum AGRP levels may act as proxy for central AGRP concentrations.

Gavrila et al. demonstrated that circulating AGRP ­levels increase during fasting in humans^23^. Obese adult males ­exhibited significantly higher AGRP plasma levels compared to their non-obese counterparts, whereas no significant difference in serum AGRP levels was observed between underweight and obese children^24,25^. A study published in 2020 compared serum AGRP levels between 88 patients with Type 2 diabetes mellitus (T2DM) and 80 healthy controls. The results indicated that serum AGRP levels were significantly lower in the T2DM group compared to the control group^26^.

To our knowledge, this is the first study in the literature to investigate serum AGRP levels in women with PCOS. However, this single-center study has several limitations. Serum AGRP levels were measured only once in the morning after an 8-h fast, and the potential impact of satiety on AGRP levels in women with PCOS was not investigated. Furthermore, the distinction between subgroups based on participants’ BMI was made using a 25 kg/m² threshold, without further differentiation between categories, such as obesity, severe obesity, and morbid obesity. The lack of analysis regarding how these varying degrees of obesity in PCOS may influence serum AGRP levels represents another notable limitation of the study. Additionally, it should be noted that these single-point serum AGRP measurements may not accurately reflect the activity of AGRP neurons in the CNS or capture dynamic fluctuations in serum AGRP levels throughout the day.

In conclusion, serum AGRP levels were assessed in groups with and without PCOS in this study. In both group and subgroup analyses, serum AGRP levels were comparable between the groups. Although the sample size is too small to draw definitive conclusions, the results suggest that serum AGRP levels are unlikely to play a significant role in the pathophysiology of PCOS or be directly affected by the condition.
